# Genetic analysis of 7 medieval skeletons from Aragonese Pyrenees

**DOI:** 10.3325/cmj.2011.52.336

**Published:** 2011-06

**Authors:** Carolina Núńez, Cecilia Sosa, Miriam Baeta, Maria Geppert, Meredith Turnbough, Nicole Phillips, Yolanda Casalod, Miguel Bolea, Rhonda Roby, Bruce Budowle, Begońa Martínez-Jarreta

**Affiliations:** 1 Department of Forensic Medicine, Faculty of Medicine, University of Zaragoza, Zaragoza, Spain; 2 Department of Forensic Genetics, Institute of Legal Medicine and Forensic Sciences, Charité-Universitätsmedizin, Berlin, Germany; 3 Department of Forensic and Investigative Genetics, University of North Texas Health Science Center, Denton, Tex, USA; 4 Institute of Investigative Genetics, University of North Texas Health Science Center, Tex, USA; * The first three authors contributed equally to this study.

## Abstract

**Aim:**

To perform a genetic characterization of 7 skeletons from medieval age found in a burial site in the Aragonese Pyrenees.

**Methods:**

Allele frequencies of autosomal short tandem repeats (STR) loci were determined by 3 different STR systems. Mitochondrial DNA (mtDNA) and Y-chromosome haplogroups were determined by sequencing of the hypervariable segment 1 of mtDNA and typing of phylogenetic Y chromosome single nucleotide polymorphisms (Y-SNP) markers, respectively. Possible familial relationships were also investigated.

**Results:**

Complete or partial STR profiles were obtained in 3 of the 7 samples. Mitochondrial DNA haplogroup was determined in 6 samples, with 5 of them corresponding to the haplogroup H and 1 to the haplogroup U5a. Y-chromosome haplogroup was determined in 2 samples, corresponding to the haplogroup R. In one of them, the sub-branch R1b1b2 was determined. mtDNA sequences indicated that some of the individuals could be maternally related, while STR profiles indicated no direct family relationships.

**Conclusions:**

Despite the antiquity of the samples and great difficulty that genetic analyses entail, the combined use of autosomal STR markers, Y-chromosome informative SNPs, and mtDNA sequences allowed us to genotype a group of skeletons from the medieval age.

The spectrum of disciplines that have the ability to detect and analyze ancient molecules has increased substantially and contributed to the capabilities of palaeobiology and genetic anthropology. The parts of human remains that are best preserved after a long period of time are bones and teeth, which are most frequently used for molecular analyses. Immediately after death and in the transition from a living organism to a fossil, some changes occur to the cadaver and its circumstances (taphonomic processes) and others take place inside the bone (diagenetic processes). The type and extent of diagenetic processes are influenced by several factors (1,2) and it has been stated that at burial all bones have similar diagenetic parameters, but when the bones are recovered these values may widely differ (1). A single parameter or combination of parameters that better predicts the degree of preservation of DNA molecules has long been sought for. Much attention has been paid to the preservation of protein in bone, mainly collagen, which is the most abundant protein in bony tissues; but there is no consensus on the possible relation of collagen with the DNA yield (3-7).

Autosomal short tandem repeats (STR) are forensically relevant genetic markers that offer the highest discrimination power and thus are the first choice for genetic identification in forensic case work. However, when it comes to ancient samples problems such as degradation, low copy number, and inhibition (8) very often preclude the analysis of relatively large fragments of nuclear DNA. Therefore, for analyzing degraded DNA, a more successful method has been mtDNA typing, due to its high copy number (1000-10 000 copies) per cell. Although autosomal STR typing is still not comparable to mtDNA typing, with the advent of highly robust commercial kits using a mini-STR format (9,10) it has become more effective than ever and is a valuable tool for molecular anthropology, archeology, and forensic genetics.

The aim of this study was to genetically characterize 7 skeletons found in a medieval burial site in the Aragonese Pyrenees, as well as to assess the performance of the currently available autosomal STR systems to genotype difficult samples.

## Materials and methods

### Samples

In a medieval burial site located in the Aragonese Pyrenees (northern Spain, latitude: 0°40’W; longitude: 42°30’N), 7 morphologically well preserved skeletons were discovered in 1985. They were buried in stone and the adjacent graves were arranged in the same layer and under similar burial conditions. There were no historical or archeological records to infer the origin of this group. The anthropological analysis revealed that all 7 were male. Two of them were around 17 years old and the rest were adults between 30 and 70 years old.

Sampling for genetic analyses was performed preferentially from the femora, although ribs were chosen from individuals that had the poorest state of general preservation as assessed macroscopically. Adjacent samples were taken for ^14^C dating.

### Radiocarbon dating (^14^C dating)

Radiocarbon dating was carried out at the Oxford Radiocarbon Accelerator Unit (ORAU, RLAHA, University of Oxford, Oxford, UK). Chemical pre-treatment, target preparation, and accelerator mass spectrometry measurement were performed according to Ramsey et al (11-13). Calibration was performed using the IntCal04 data set (14).

### DNA analyses

*Prevention of contamination.* In order to minimize contamination by exogenous DNA, some of the generally accepted guidelines for aDNA studies were followed (15). Anthropologists and laboratory staff were genotyped and their profiles were compared with those of the samples. All laboratory staff were women. Appropriate laboratory clothing was used by all the analysts. All procedures were carried out under maximum sterile conditions in a dedicated laboratory with separated areas for specimen handling, pre-polymerase chain reaction (PCR), and post-PCR analyses. DNA-free certified reagents were used, and materials and solutions were properly decontaminated (UV exposure/autoclaving). All working surfaces were frequently treated with commercial bleach. Multiple reagent blanks and negative control reactions were performed with all amplifications. Bone extraction was performed in two independent laboratories using different methods. Moreover, a minimum of 3 amplifications were carried out from different extracts of the same sample to increase the confidence in the results.

*DNA extraction and quantification.* Before sampling and in order to eliminate surface contamination, a 1-2-mm layer of the outer surface of the bones was removed by sanding, and transversal hemi-sections were cut with a diamond disc attached to a hand drill. Afterwards the samples were thoroughly cleaned (with successive washes of 10% sodium hypochlorite, ethanol, and sterile water), and pulverized under liquid nitrogen in a freezer mill. Samples were prepared and extracted individually.

Bone powder (up to 500 mg) was demineralized overnight using 0.5 M EDTA. After centrifugation, DNA from the supernatant was extracted by two silica-based methods: either the one described by Rohland and Hofreiter (16) with minor modifications (17) or by large volume columns described by Turnbough et al (18). At least 3 independent DNA extracts were performed. Mock extractions were carried out along with bone samples for each method to monitor contamination.

The quantity of the extracted DNA was determined by real time PCR kit Quantifiler^®^ Human DNA Quantification Kit (Applied Biosystems, Foster City, CA, USA). The kit includes an internal PCR control to monitor inhibition of the PCR.

*Autosomal STR analysis.* Autosomal STR loci were studied using AmpFiSTR Identifiler^®^ Plus PCR Amplification Kit (Applied Biosystems). In addition, 2 samples that yielded complete profiles were also analyzed with AmpFiSTR^®^ NGM PCR Amplification Kit (Applied Biosystems). Mini-STR loci were typed using the AmpFiSTR^®^ MiniFiler PCR Amplification Kit (Applied Biosystems). The amount of DNA used for genotyping ranged from about 0.1 ng to 1 ng. Thermal cycling conditions were performed according to the manufacturer’s recommendations for all kits. However, in certain cases, the number of cycles was increased to 32 (instead of 29 for Identifiler^®^ Plus and 30 for MiniFiler^TM^).

A minimum of 3 amplifications were carried out from different extracts from the same sample, and the PCR products were analyzed at least twice. From all the results of each sample, consensus alleles were determined when alleles were observed in at least two replicates.

*Mitochondrial DNA analysis.* Mitochondrial DNA hypervariable segment one (HVS1) was analyzed. PCRs contained 2.5 µL 10 × PCR Buffer II, 2 µL MgCl_2_ (25 mM), 1 µL dNTP mix (10 mM), 2.5 µL BSA, 0.5 µL of each primer (10 mM), 0.5 µL AmpliTaq Gold (5 U/µL), 10 µL of DNA sample, and sterile water up to a total volume of 25 µL. Two sets of primers were used to cover the amplification of the whole HVS1: a) primers A1 (5′-CACCATTAGCACCCAAAGCT-3′) and B1 (5′-GAGGATGGTGGTCAAGGGAC-3′) producing an amplicon of 432 bp; b) primers A1 and B2 (5′-GGCTTTGGAGTTGCAGTTGAT-3′), A2 (5′-TACTTGACCACCTGTAGTAC-3′) and B1 were used to generate 2 overlapping fragments of 279 and 270 bp, respectively (19). Cycling parameters were 95°C for 11 minutes, followed by 36 cycles of 95°C for 10 seconds, 61°C for 30 seconds, and 72°C for 30 seconds, and a final extension of 70°C for 10 minutes. Amplification products were subjected to electrophoresis on 2% agarose mini-gels and visualized with ethidium bromide and UV light. DNA products were purified using 5 µL of ExoSAP-IT^®^ (USB Corporation, Cleveland, OH, USA) to 25 µL of PCR product and subsequently sequenced in both directions using the PCR primers. Extension reactions were performed by BigDye^®^ Terminator^TM^ v1.1 Cycle Sequencing Kit (Applied Biosystems). A reaction included 1 µL BigDye^®^ Terminator^TM^ mix, 5 µL BetterBuffer (The Gel Company, San Francisco, CA, USA), 1.5 µL of each primer (3.3 µM), and 7.5 µL of the PCR product in a final volume of 15 µL. Manufacturer’s cycle sequencing parameters were followed. Sequencing products were purified with BigDye^®^ XTerminator^TM^ Purification Kit (Applied Biosystems) following the manufacturer’s recommendations. Capillary electrophoresis was carried out on the 3130xl Genetic Analyzer (Applied Biosystems), and sequencing reaction products were analyzed with the Sequence Scanner^TM^ v1.0 (Applied Biosystems). Sequences were aligned to the revised Cambridge Reference Sequence (rCRS) (20) using ClustalW (21) and edited from np 16050 to 16390.

Haplogroups were assigned following established rules and definitions (22,23) and the most updated version of the mtDNA phylogeny (24). Haplotype frequencies were obtained from the European sample populations within the EDNAP mtDNA Population Database (EMPOP) database (25).

In order to minimize the effects of potential laboratory and transcription errors, the data were analyzed separately by 3 independent analysts and evaluated based on known phylogeny. Extraction and genotyping were repeated at least 3 times, and consensus sequences were obtained from the replicates.

*Y-SNPs typing.* Samples from femur 1 and 3, which were successfully analyzed in the previous analyses, were selected for Y-SNP genotyping. First, samples were tested with the Multiplex Major described by Geppert et al (26), which includes the 12 Y-SNPs (M42, M207, M242, M168, M3, M145, M174, M213, RPS4Y711, M45, P170, and M9) defining the most frequent major haplogroups. The multiplex analysis was performed using 400-500 pg of DNA.

The multiplex single base extension (SBE) reaction was performed as described by Geppert et al (26), with slight modifications, in a total volume of 6 µL containing 1.5 µL ABI PRISM^®^ SNaPshot^TM^ Multiplex Ready Reaction Mix (Applied Biosystems), 0.42 µM SBE primer, 2 µL cleaned-up PCR product, and ultrapure water. The SBE reaction was cycled under the following conditions: 96°C for 10 seconds, 50°C for 5 seconds, and 60°C for 30 seconds, for 25 cycles. The SBE reaction was cleaned up with 1 U of shrimp alkaline phosphatase and by incubating the reaction at 37°C for 60 minutes followed by 85°C for 15 minutes.

Single amplifications were also carried out for the following SNPs with shorter amplicons: CT-M168, I-M170, R-M207, R1b1b2-M269, and R1a1a-M198. Singleplex PCR was performed in a total volume of 12 µL with 0.8 µM of each primer, 0.25 µM dNTPs, 1 U Gold Taq, 1 × PCR-Puffer, 1µM MgCl_2_, 300-400pg DNA, and ultrapure water. PCR was conducted with the following protocol: 95°C for 10 minutes followed by 8 cycles of 95°C for 30 seconds, 60°C for 30 seconds, 72°C for 30 seconds, 40 cycles of 95°C for 30 seconds, 58°C for 30 seconds, 72°C for 30 seconds, and a final extension of 72°C for 5 minutes. The PCR was cleaned up with 2 µL of ExoSAP-IT (USB) per 5 µL reaction volume and incubated for 60 minutes at 37°C followed by 15 minutes at 75°C. SBE reaction was performed according to the protocol given for the multiplex assay, but with single primers. Purification of the SBE reaction was performed with 1 µL SAP (USB) per reaction.

Capillary electrophoresis of the SBE fragments was run on an ABI Prism Genetic Analyzer (3130xl; Applied Biosystems) and the data were analyzed using GeneMapper^®^ ID Analysis software v3.2.

## Results

Radiocarbon dating indicated that the skeletons dated from the years 776 to 991 AD (Table 1). DNA yields varied among samples (Table 1). In general, there was no noticeable PCR inhibition, except for minor inhibition with rib 2.

**Table 1:** Radiocarbon dates and DNA yield of the samples from 7 medieval skeletons from the Aragonese Pyrenees

Sample	Uncalibrated radiocarbon years BP*	Calendar age ranges^†^	DNA yield^‡^
Rib 1	1119 ± 24	885-987	1.2
Femur 1	1171 ± 26	776-900	10.0
Femur 2	1121 ± 23	885-985	0.6
Femur 3	1147 ± 26	807-974	6.0
Femur 4	1105 ± 25	889-991	0.1
Rib 2	1123 ± 26	870-990	1.3
Femur 5	1111 ± 26	885-991	0.1

*Before present – 1950.

†Radiocarbon dates are uncalibrated years BP.

**‡**DNA yield is expressed in nanograms per gram of bone powder (ng/g).

### Autosomal STR analyses

Only 3 of the 7 samples yielded complete or partial genetic profiles with Identifiler^®^ Plus and MiniFiler^TM^ (Table 2). Femur 1 and 3 yielded complete profiles with Identifiler^®^ Plus and partial profiles with MiniFiler^TM^. Femur 5 yielded partial profiles with both multiplex kits. Additionally, femur 1 and 3 were analyzed using NGM^TM^ PCR Amplification Kit and both obtained partial profiles. The amelogenin marker was typed successfully in 4 of the 7 samples (femur 1, 2, 3, and 5), indicating that they belonged to male individuals, which was consistent with the anthropological exam results.

**Table 2:** Consensus genetic profiles of Identifiler^®^ Plus, MiniFiler^TM^, and NGM^TM^ from samples analyzed in this study. An empty cell indicates that the locus was not present in the autosomal short tandem repeat kit in the studied genetic system. A dash (-) indicates no results for that locus

	Femur 1			Femur 3			Femur 5	
LOCUS	IDENTIFILER PLUS	MINIFILER	NGM	IDENTIFILER PLUS	MINIFILER	NGM	IDENTIFILER PLUS	MINIFILER
D3S1358	14, 15		14,15	15, 17		15,17	13, 14	
D19S433	14/-		14/-	13, 15		-	16/-	
D8S1179	12, 13		12,13	10, 15		10,15	13, 14	
D5S818	11, 12			12, 13			12/-	
TH01	8, 9.3		8,9.3	8, 9		-	-	
vWA	18/-		18/-	16, 17		-	14/-	
D21S11	31.2, 32.2	-	31.2/-	30, 32.2	30, 32.2	-	-	32.2/-
D13S317	8, 11	8, 11		13, 14	13, 14		8, 13	8, 13
TPOX	8/-			8, 10			-	
D7S820	11/-	10, 11		10, 13	10, 13		-	-
D16S539	11, 14	11, 14	11,14	11/-	-	-	12, 14	-
D18S51	13, 17	13, 17	13/-	12, 13	12, 13	-	-	16/-
CSF1PO	10, 11	10, 11		9, 11	9, 11		-	12, 13
D2S1338	17, 24	17, 24	17/-	20, 27	20, 27	-	-	16, 17
FGA	21/-	-	21,25	21, 24	21, 24	-	22/-	22, 25
D10S1248			14,15			13,14		
D22S1045			11,15			11,16		
D2S441			10,11.3			11.3,14		
D1S1656			12,16			-		
D12S391			18,19.3			-		
Amel	XY	XY	XY	XY	XY	XY	XY	XY

For the 3 samples that yielded autosomal STRs results (femur 1, 3, and 5), likelihood ratios (LR) were calculated for different hypotheses in order to investigate possible familial relationships. STR markers showing homozygosity were considered as potential allelic drop-outs and were not included in the analyses. No direct relationships (fatherhood or other paternal relationships) were detected, although other familial relationships could not be excluded.

### mtDNA analysis

Sequence variation in the HVS1 was investigated in all samples. Reproducible HVS1 sequences were obtained for all the samples except for rib 2, which did not produce conclusive results (Table 3). Two different mtDNA haplotypes were observed twice in our samples. Femur 1 and 5 shared haplotype 16069T 16126C 16300G, while femur 4 and rib 1 showed no differences from the rCRS for the analyzed range (16050-16390). Most of the mtDNA types were assigned to the haplogroup H and 1 sequence (from femur 3) to the haplogroup U5a. The frequency for the haplotype equal to the rCRS shared by femur 4 and rib 1 in European sample populations included in the EMPOP database was significantly high (*P* = 0.1167). On the other hand, the frequency of the haplotype 16069T 16126C 16300G shared by femur 1 and 5 was low (*P* = 0.00045). This frequency corresponds to a LR of 2222, meaning that it is 2222 times more probable that these individuals are maternally related than that they are not related. The LR for mtDNA and STRs was combined and the highest value (LR = 1769) was obtained, favoring the hypothesis that they were maternally related cousins.

**Table 3:** mtDNA hypervariable segment 1 sequences (range 16050-16390) of 6 specimens under study. Dots indicate no difference from the revised Cambridge reference sequence

Sample	Haplogroup	16069	16126	16192	16256	16270	16291	16300
Femur 1	H	T	C	.	.	.	.	G
Femur 2	H	.	.	.	.	.	T	.
Femur 3	U5a	.	.	T	T	T	.	.
Femur 4	H	.	.	.	.	.	.	.
Femur 5	H	T	C	.	.	.	.	G
Rib 1	H	.	.	.	.	.	.	.

### Y-SNPs analysis

Results of the Y-SNPs analysis are shown in Table 4. Multiplex Major, including 12 Y chromosome SNPs defining the major Y chromosome haplogroups, was studied in the femur 1 and 3. This analysis failed for femur 3, while for femur 1 one Y-SNP marker was amplified successfully – M42 that defines the large cluster of haplogroup B-T. Concerning this result, the approach of using a multiplex assay to type the samples was rejected and single amplifications, using SNP markers with the short amplicons (CT-M168, I-M170, R-M207, R1b1b2-M269, and R1a1a-M198) were set up in single PCRs. Results showed that femur 1 could be assigned to the haplogroup R1b1b2, which is defined by the marker M269. On the other hand, femur 3 could be assigned to the haplogroup R, which is defined by the markers M207, M306, P224, P227, P229, P232, P280, and P285 (M207 was tested). It was not possible to obtain further subtyping within branch R, beyond the exclusion of all sub-branches of R1a1a, which is defined by the Y-SNP M198 (that was tested to be ancestral).

**Table 4:** Y-chromosome single nucleotide polymorphisms (Y-SNP) typing results for femur 1 and 3. A plus sign indicates the derived status of the SNP and a minus sign indicates ancestral status

Sample	Multiplex Major	M168 (CT)	M207 (R)	M170 (I)	M198 (R1a)	M269 (R1b1b2)	Haplogroup
Femur 1	M42 (+)	+	analysis failed	-	-	+	R1b1b2
Femur 3	analysis failed	+	+	-	-	analysis failed	R

None of the STR profiles, mtDNA haplogroups, or Y-SNPs matched those of the people who handled the samples (ie, laboratory staff, anthropologists).

## Discussion

This study presents DNA typing of bone remains belonging to 7 male skeletons anthropologically individualized and carbon dated to the period 776-991 year AD. When DNA typing was successful, morphological and genetic sex assessments were concordant. All individuals were male, 5 were adults aged between 30 and 70 years and 2 were around 17 years old.

In spite of the fact that the skeletons were buried under similar conditions, different degrees of preservation were observed during the anthropological examination, generally consistent with different DNA yields. Accordingly, different samples showed varying degrees of genetic information. No fatherhood or other paternal relationships were demonstrated among them.

Confidence in authenticity is always a concern in human ancient DNA studies, and strict measures have been suggested to increase the reliability of the study (15,27), as well as to perform a self-critical and rational analysis of the data (28,29). The following criteria speak in favor of the authenticity of the results presented here: a) careful procedures were observed for specimen handling and preparation, b) clean negative controls and reagent blanks were used, c) reproducible and unambiguous genetic profiles were obtained in different laboratories (no mixtures were found), d) the same profiles were obtained from different samples of the same skeleton, e) a different genetic profile was observed in every skeleton typed, e) there was a concordance of morphological and genetic sexing, and f) the greatest amplification efficiency of shorter STRs was observed.

It is not surprising that the samples that gave the greatest DNA quantity (femur 1 and 3) also presented complete profiles with Identifiler^®^ Plus. However, when amplified with the NGM^TM^ kit, only femur 1 yielded a complete profile, whereas femur 3 yielded results only for the shortest STR markers. Seven of the 10 non-amplified markers were shared with the Identifiler^®^ Plus results. Given that they have similar sizes in both multiplexes, a possible explanation could be that different extracts had DNA with different degrees of degradation. Additionally, it can be suggested that Identifiler^®^ Plus may provide better amplification efficiency than NGM^TM^ when dealing with difficult samples. It is striking that femur 5, in spite of having had a low DNA yield, produced a partial profile with MiniFiler^TM^ and Identifiler^®^ Plus.

Two mtDNA haplogroups were observed based on HVS1 sequences in our samples, H and U5a. The haplogroup composition of the studied individuals fit well in the European pattern. The haplogroup H, which is the most common in our sample (5 out of 6 typeable individuals), is widely present in European populations with frequencies above 30% (30-34), including the contemporary population of Spain (35,36). When evaluating haplogroup H sub-haplogroups, it was observed that frequency distributions of sub-haplogroups H1 and H3 demonstrated frequency peaks both centered in Iberia and the surrounding areas (33). Thus, further mtDNA analysis by HVS2 sequencing and the study of coding region SNPs would be valuable in order to resolve deeper lineages within the haplogroup H.

Femur 3 was assigned to mtDNA haplogroup U5a. The most ancient mitochondrial haplogroup U5 is found in northern and southern Europe, whereas U5a is mainly restricted to southern Europe, with some diverged individuals present in the northwest (22). It has been argued that expansions of U5-subclusters, particularly U5a and U5b, occurred after the last glacial maximum period, during re-occupation of large areas of northern Europe by refugees from the Pyrenees region (37). The frequency of haplogroup U5a in Spanish populations is approximately 8% (35).

It should be noted that there was a match between femur 1 and 5, and femur 4 and rib 1, indicating that these two pairs of individuals may be maternally related.

HVS1 data do have a limited power of discrimination in a forensic (or anthropological) context and thus many mtDNA haplogroups are poorly defined by the control region. Since only the HVS1 region was analyzed, sharing the same HVS1 profile does not necessarily imply that two mtDNAs absolutely belong to the same haplogroup (38).

The Y chromosome haplogroup R determined for femur 1 and 3 is in agreement with the geographical origin of the studied human remains. The majority of European Y chromosomes belong to this clade (39-41). Particularly, the sub-haplogroup R1b1b2, determined in femur 1, occurs at a high frequency throughout western Europe (according to *www.YHRD.org*).

This genetic study is part of an interdisciplinary research project that integrates the investigations of historians, archeologists, and anthropologists. There is of yet no historical or archeological evidence to infer the origin of this human group, so integration of different approaches may allow a better understanding of the origin of these people and possibly of the circumstances of their death and burial.

In summary, the case presented here shows that despite the antiquity of the samples, the combined use of current common forensic genetic systems allows the retrieval of valuable and interesting genetic information. With greater sensitivity of detection methods and more human identity genetic markers that are amenable to analysis of degraded samples, more challenging anthropological samples may disclose the secrets of their genetic make-up.
